# Differential Requirement for c-Jun N-terminal Kinase 1 in Lung Inflammation and Host Defense

**DOI:** 10.1371/journal.pone.0034638

**Published:** 2012-04-13

**Authors:** Jos Van der Velden, Yvonne M. W. Janssen-Heininger, Sivanarayna Mandalapu, Erich V. Scheller, Jay K. Kolls, John F. Alcorn

**Affiliations:** 1 Department of Pathology, University of Vermont, Burlington, Vermont, United States of America; 2 Department of Pediatrics, Children's Hospital of Pittsburgh of UPMC, Pittsburgh, Pennsylvania, United States of America; 3 RK Mellon Foundation Institute, Children's Hospital of Pittsburgh of UPMC, Pittsburgh, Pennsylvania, United States of America; French National Centre for Scientific Research, France

## Abstract

The c-Jun N-terminal kinase (JNK) - 1 pathway has been implicated in the cellular response to stress in many tissues and models. JNK1 is known to play a role in a variety of signaling cascades, including those involved in lung disease pathogenesis. Recently, a role for JNK1 signaling in immune cell function has emerged. The goal of the present study was to determine the role of JNK1 in host defense against both bacterial and viral pneumonia, as well as the impact of JNK1 signaling on IL-17 mediated immunity. Wild type (WT) and JNK1 −/− mice were challenged with *Escherichia coli*, *Staphylococcus aureus*, or Influenza A. In addition, WT and JNK1 −/− mice and epithelial cells were stimulated with IL-17A. The impact of JNK1 deletion on pathogen clearance, inflammation, and histopathology was assessed. JNK1 was required for clearance of *E. coli*, inflammatory cell recruitment, and cytokine production. Interestingly, JNK1 deletion had only a small impact on the host response to *S. aureus*. JNK1 −/− mice had decreased Influenza A burden in viral pneumonia, yet displayed worsened morbidity. Finally, JNK1 was required for IL-17A mediated induction of inflammatory cytokines and antimicrobial peptides both in epithelial cells and the lung. These data identify JNK1 as an important signaling molecule in host defense and demonstrate a pathogen specific role in disease. Manipulation of the JNK1 pathway may represent a novel therapeutic target in pneumonia.

## Introduction

Bacterial and viral pneumonia represents a significant cause of morbidity and mortality worldwide. Bacterial pneumonia is a commonly encountered lung infection in both hospital acquired and community acquired settings. Infection with either gram negative or gram positive bacteria results in lung inflammation, tissue damage, and in some cases life-threatening sepsis. Despite numerous antibiotic therapies, these infections often result in poor patient outcomes. Influenza A infection by either seasonal or pandemic virus is of increasing epidemiologic importance in recent years. Influenza A infection results in lung cell apoptosis, injury, and remodeling. In worst cases, severe inflammation and co-infection may lead to mortality. Anti-viral therapies are effective at reducing viral burden; however, lung injury often persists. The need for identification of novel pathways in pneumonia pathogenesis is great in order to design new therapeutic approaches. Many cellular signaling pathways have been investigated for their role in these processes.

The MAPK family member c-Jun N-terminal kinase (JNK) comprises three members, JNK1–3, with numerous alternate splicoforms [1]. JNK1 and JNK2 are ubiquitously expressed, while JNK3 is restricted to the brain, testes, and heart. JNK1 is known to play a role in cellular stress responses, apoptosis [2], and was recently shown to modulate lung remodeling following injury [3]–[5]. The JNK1 signaling pathway is complex and its roles in both innate and adaptive immune responses have been recently reviewed [1]–[2], [6]–[7]. A primary consequence of JNK1 activation, via phosphorylation by upstream kinases, is phosphorylation of AP-1 transcription factors, including c-Jun. In this manner JNK1 plays an important role in transcriptional regulation in response to a number of stimuli. JNK1 is activated by the gram-negative bacterial component lipopolysaccharide (LPS) via TLR4 [8]–[9] and JNK1 is required for chemokine production by macrophages [10]–[11]. These data suggest an important role for JNK1 in innate immune responses. JNK1 has also been shown to play a role in regulating helper T cell function. Naïve CD4^+^ T cells express low levels of JNK1 and JNK2, however upon activation, these proteins are highly upregulated and display increased activity [1]#. These data define an emerging role for JNK in both innate and adaptive immunity.

The goal of this study was to investigate the role of JNK1 in host defense against bacterial and viral pneumonia. In addition, the potential immunologic mechanism by which JNK1 interacts was examined. IL-17A has been implicated in host defense against many pathogens, both intra- and extra-cellular in nature. The impact of JNK1 on IL-17A signaling was also addressed. Since many prior studies evaluating the role of JNK1 in inflammation have utilized non-specific pharmacologic inhibitors in cell lines, these studies were conducted utilizing JNK1 −/− mice and primary epithelial cells from mice lacking JNK1.

## Results

### JNK1 regulates lung inflammation in bacterial pneumonia

**Figure 1:**
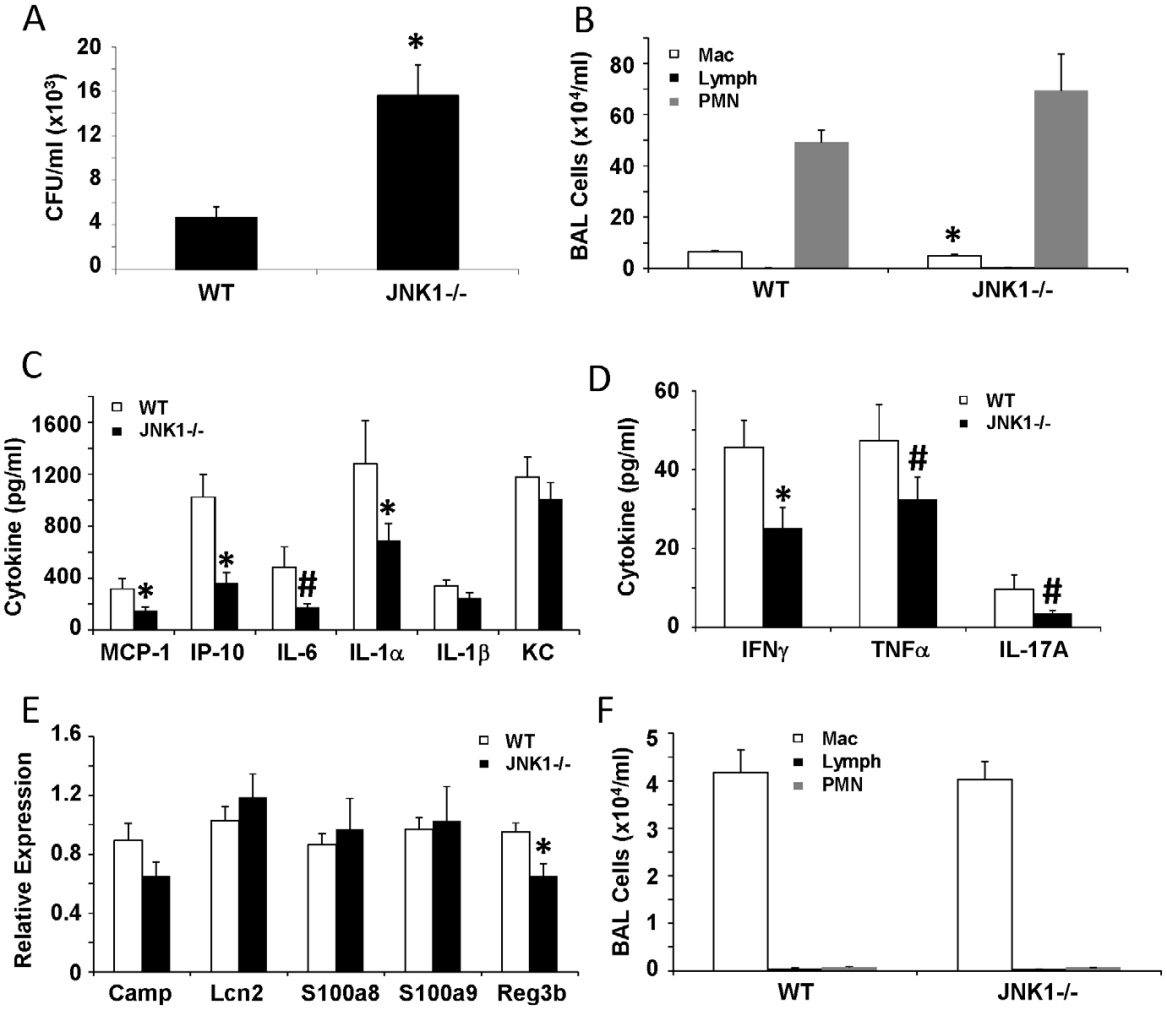
JNK1 is required for host defense against gram-negative bacterial pneumonia. WT and JNK1 −/− mice were challenged with 10^7^ cfu of *E. coli* for 24 hours (N = 8, 10). A – bacterial colony counts in lung homogenate. B – inflammatory cells in BAL fluid. C, D – inflammatory cytokines in lung homogenate. E – antimicrobial peptide expression in lung tissue. F – inflammatory cells in BAL fluid from mice challenged with PBS for 24 hours (N = 6, 6). * p<0.05, # p<0.10.

**Figure 2:**
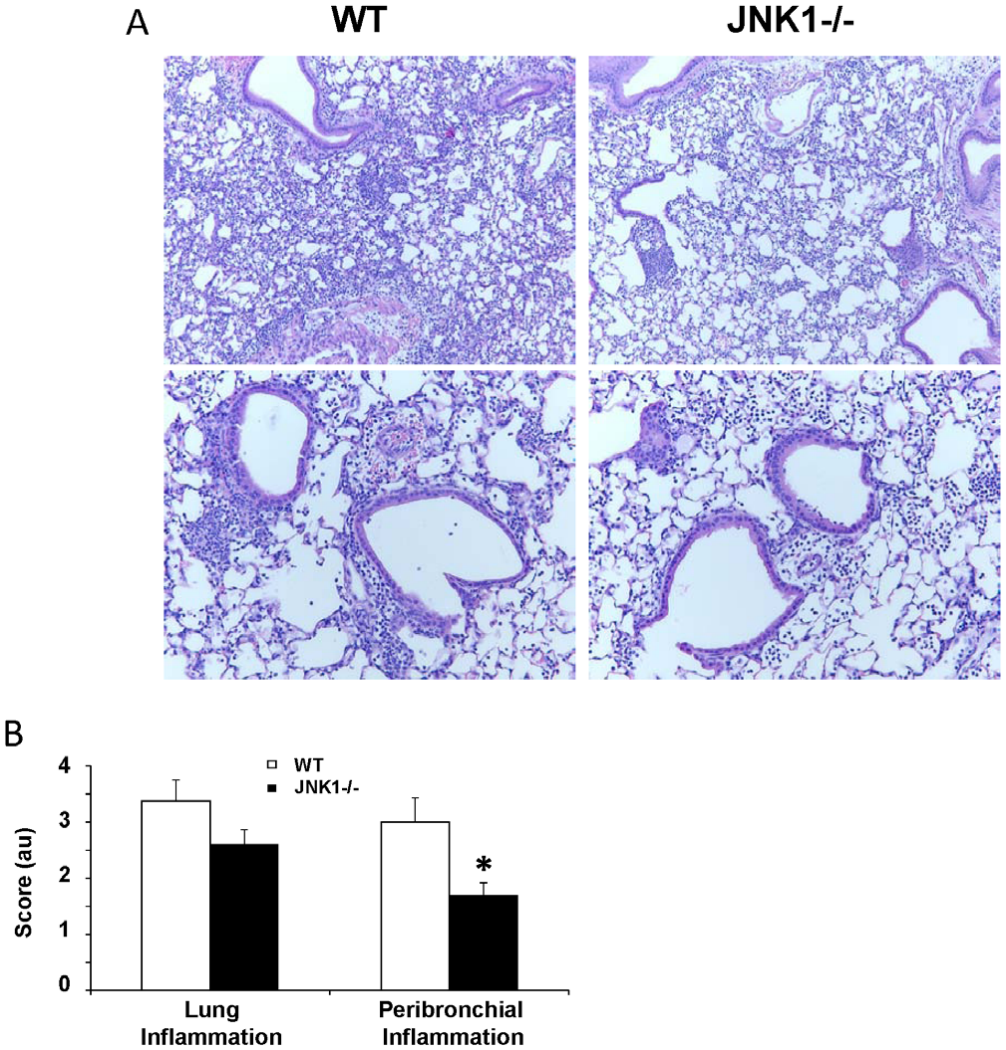
JNK1 is required for peribronchial inflammation during gram-negative bacterial pneumonia. WT and JNK1 −/− mice were challenged with 10^7^ cfu of *E. coli* for 24 hours (N = 8, 10). A – lung histology 100× (top panels), 200× (lower panels). B – lung histology scoring. * p<0.05.

JNK1 is known to modulate numerous responses to cellular stress including inflammatory stimuli. The majority of studies addressing the role of JNK1 in host defense have utilized non-specific inhibitor approaches, often employing in vitro approaches. To examine whether JNK1 is required for bacterial host defense in vivo, we challenged WT and JNK1 −/− mice with the gram-negative bacterium *E. coli*. JNK1 −/− mice displayed a nearly four-fold increase in bacterial burden in the lung one day after challenge (Figure 1A, B). While total inflammatory cell recruitment in BAL was not different, the profile of cells in JNK1 −/− mice was characterized by significantly less macrophages than WT mice. To further examine the impact of JNK1 deletion on inflammation, we examined lung histopathology. JNK1 −/− mice had significantly decreased peribronchial inflammation compared to WT mice (Figure 2). JNK1 −/− mice trended towards having reduced overall lung parenchymal inflammatory cellular infiltrates. Next, the effect of JNK1 depletion on cytokine induction was examined by cytokine multiplex assay. JNK1 −/− mice produced significantly less MCP-1, IFNγ, IP-10, and IL-1α versus WT mice (Figure 1C, D). In addition, JNK1 −/− mice had a trend towards decreased IL-6, TNFα, and IL-17A production. Since IL-23 is required for IL-17A production, we measured IL-23p19 in the lung homogenate. WT and JNK1 −/− mice produced similar levels of IL-23p19 (242.9±39.3 pg/ml vs. 250.5±28.8 pg/ml, respectively) in response to *E. coli* challenge. We then examined whether JNK1 was required for antimicrobial peptide production in response to *E. coli*. JNK1 −/− mice produced significantly less Reg3b and a trend towards less Camp compared to WT mice (Figure 1E). Finally, to confirm that the difference in cellular inflammation between WT and JNK1 −/− mice was not due to baseline differences between the mice, WT and JNK1 −/− mice were challenged with PBS for 24 hours. Differential BAL cell counts showed no changes in macrophage numbers between the mouse strains (Figure 1F). These data indicate that JNK1 is required for the normal immune response to the gram-negative bacteria *E. coli*. Deletion of JNK1 resulted in decreased lung inflammation and increased pathogen burden.

**Figure 3:**
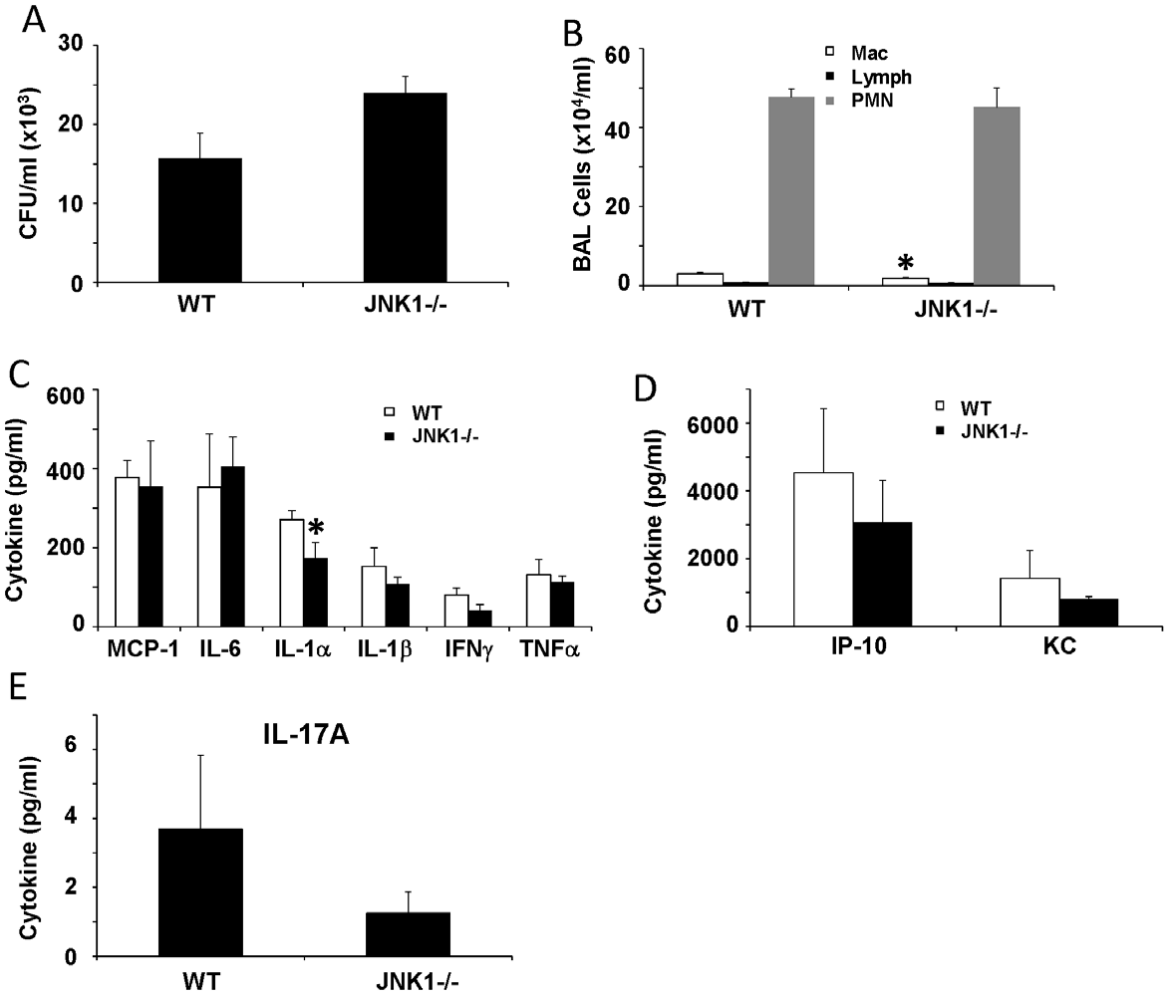
JNK1 is not required for host defense against gram-positive pneumonia. WT and JNK1 −/− mice were challenged with 10^8^ cfu of *S. aureus* for 24 hours (N = 5, 8). A – bacterial colony counts in lung homogenate. B – inflammatory cells in BAL fluid. C, D, E – inflammatory cytokines in lung homogenate. * p<0.05.

*E. coli*, and other gram-negative bacteria, drives inflammation through interaction of LPS with the Tlr4 signaling cascade [12]. Gram-positive bacteria initiate inflammation largely through interactions with Tlr2 and other pathways [13]. To test whether JNK1 plays a role in host defense against gram-positive bacteria, we challenged WT and JNK1 −/− mice with *S. aureus*. JNK1 −/− mice did not have significantly elevated *S. aureus* burden one day after challenge (Figure 3). Similar to the *E. coli* challenge model, JNK1 −/− and WT mice had similar BAL cell numbers, but JNK1 −/− mice recruited significantly less macrophages. Deletion of JNK1 resulted in significantly less IL-1α production, but did not impact other cytokines that were decreased in the gram-negative model. These data suggest that JNK1 does not play a large role in host defense or inflammation in response to the gram positive bacterium *S. aureus*.

### JNK1 modulates the pathophysiology of Influenza A infection

**Figure 4:**
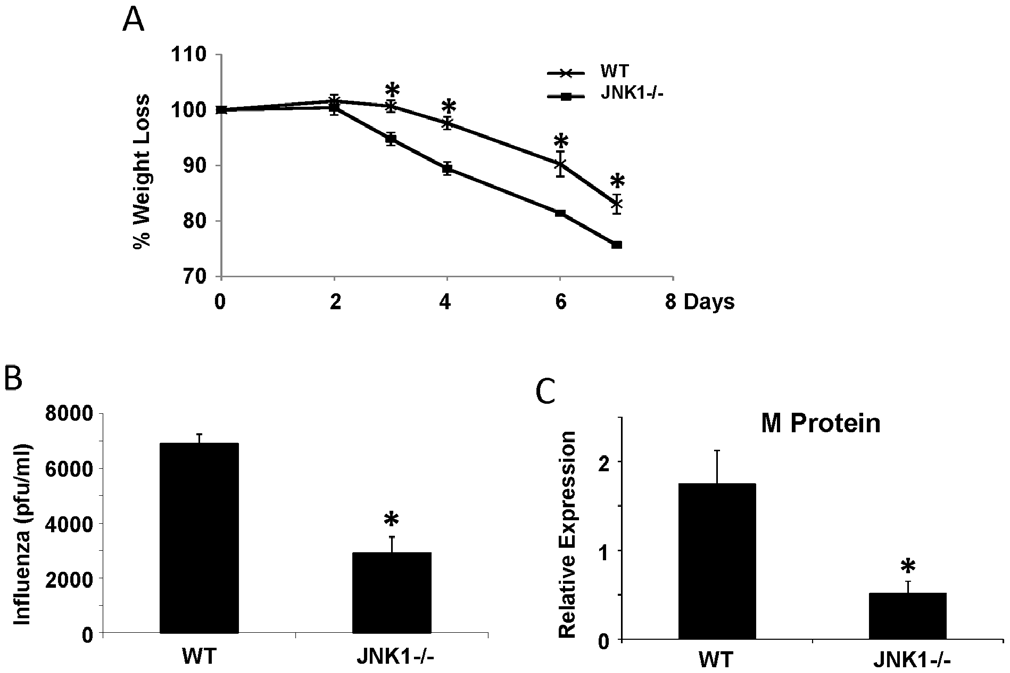
JNK1 regulates host defense against Influenza A infection. WT and JNK1 −/− mice were challenged with 150 pfu of Influenza A PR/8/34 H1N1 for 7 days. A – weight loss during infection (N = 9, 8). B – viral burden by plaque assay (N = 4, 4). C – viral burden by RT-PCR for M protein expression (N = 9, 8). * p<0.05.

**Figure 5:**
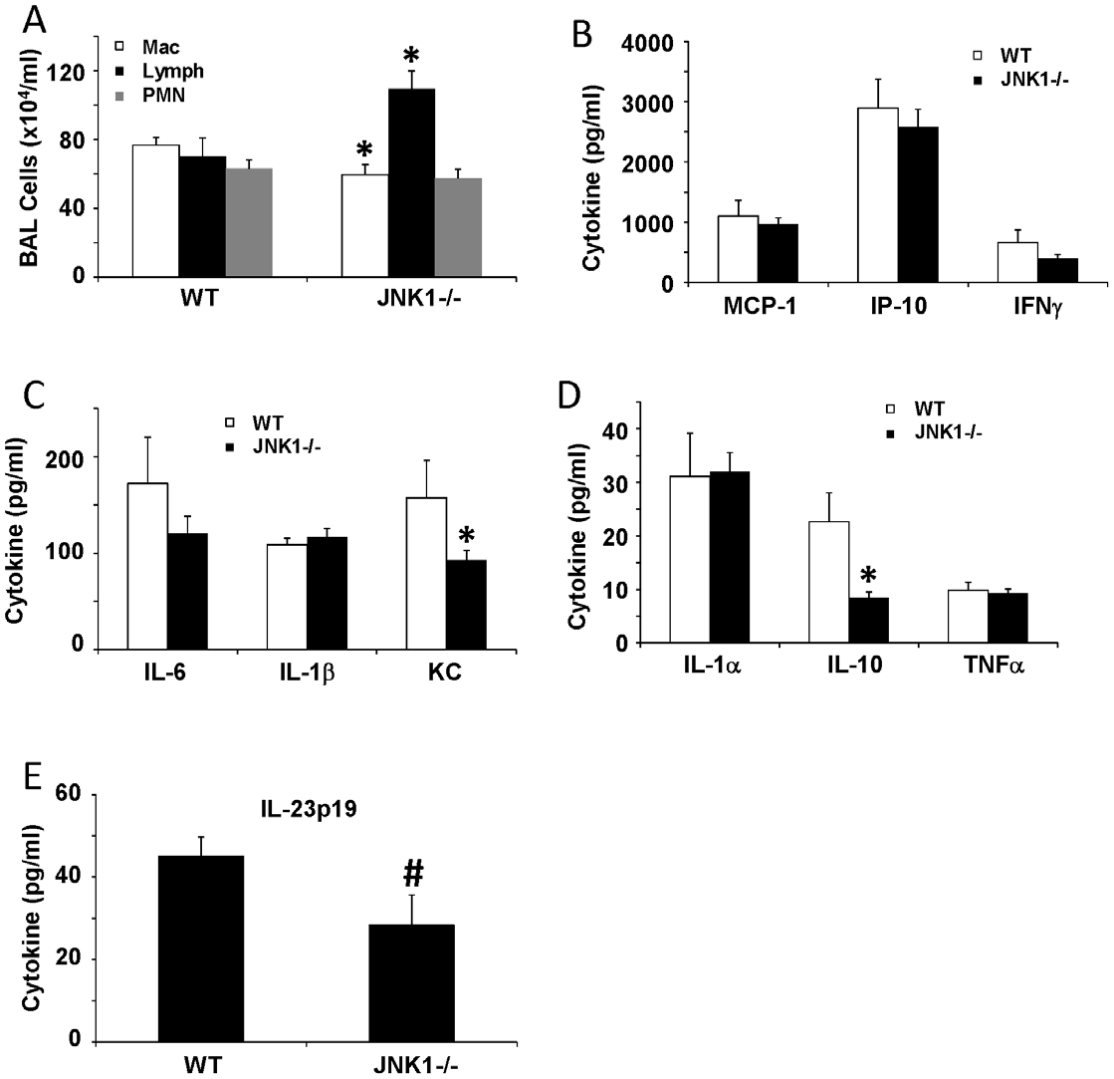
JNK1 modulates Influenza A induced inflammation. WT and JNK1 −/− mice were challenged with 150 pfu of Influenza A PR/8/34 H1N1 for 7 days. A – inflammatory cells in BAL fluid. B, C, D, E – inflammatory cytokines in lung homogenate. * p<0.05, # p<0.10.

**Figure 6:**
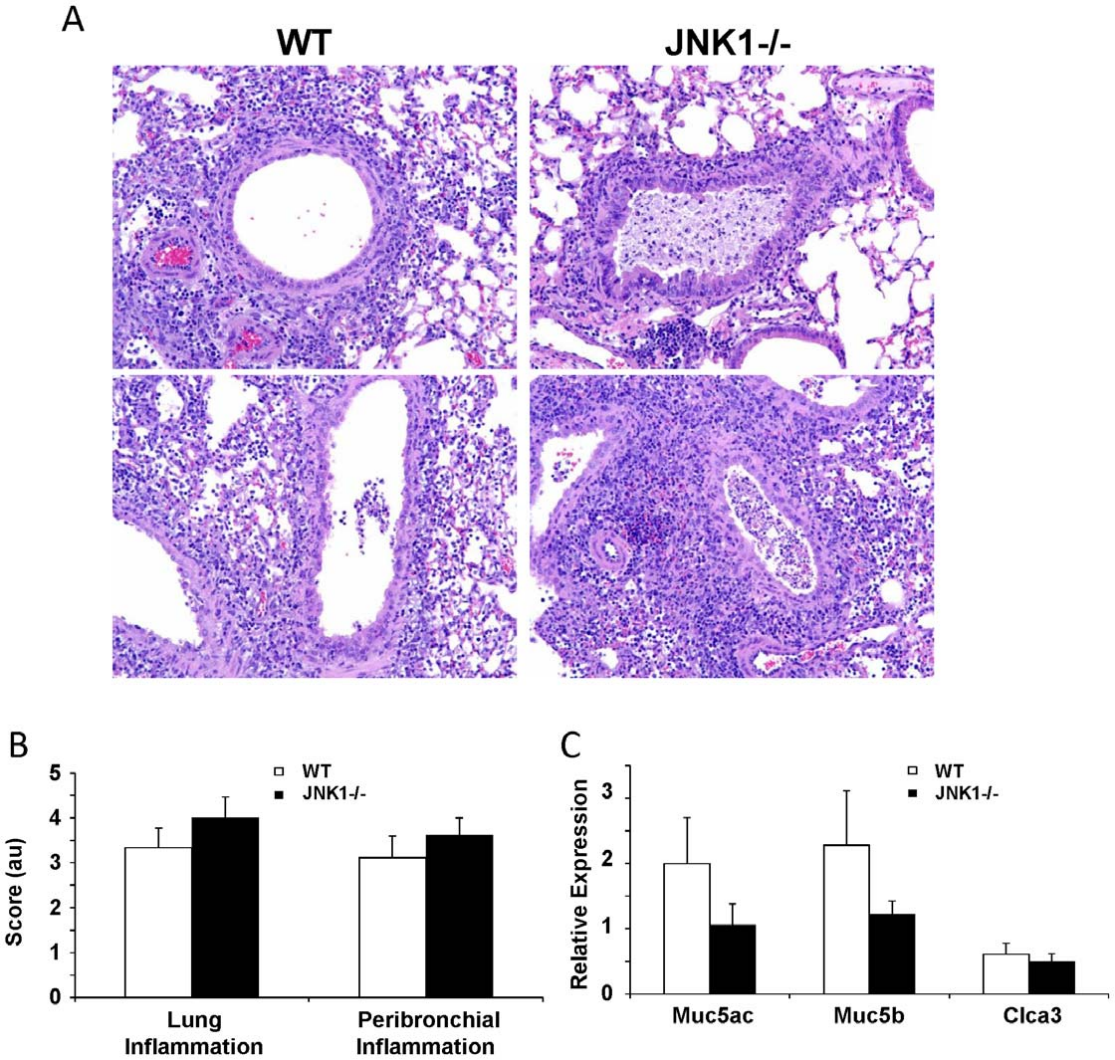
JNK1 alters lung histopathology during Influenza A infection. WT and JNK1 −/− mice were challenged with 150 pfu of Influenza A PR/8/34 H1N1 for 7 days. A – lung histology 200× (N = 9, 8). B – lung histology scoring (N = 9, 8). C – mucin gene expression by RT-PCR (N = 9, 8).

**Figure 7:**
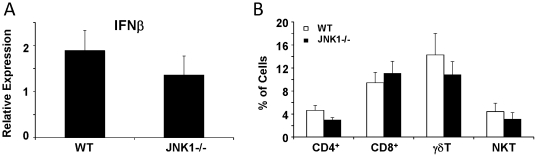
JNK1 is not required for antiviral interferon or T cell recruitment in response to Influenza A. WT and JNK1 −/− mice were challenged with 150 pfu of Influenza A PR/8/34 H1N1 for 7 days. A – type I interferon expression by RT-PCR (N = 9, 8). B – T cell profile in lung homogenate by flow cytometry (N = 7, 9).

Studies presented thus far addressed the role of JNK1 in host defense against extracellular pathogens. Next, the role of JNK1 in intracellular host defense was evaluated. WT and JNK1 −/− mice were infected with Influenza A PR/8/34 H1N1 for seven days. JNK1 −/− mice displayed increased weight loss throughout the infection time course compared to WT mice (Figure 4A). Interestingly, despite having greater morbidity as measured by weight loss, JNK1 −/− mice had decreased viral burden (measured by viral matrix protein expression) versus WT mice on day seven (Figure 4B, C). The total number of BAL inflammatory cells was unaltered in JNK1 −/− mice, however, these mice had significantly decreased macrophage recruitment and increased lymphocyte numbers compared to control mice (Figure 5A). One possible explanation for increased morbidity would be an enhanced inflammatory profile or cytokine storm in JNK1 −/− mice. Analysis of tissue inflammation by histopathology revealed no differences in parenchymal or peribronchial inflammation (Figure 6A, B). Consistent with the small changes in inflammation observed, JNK1 −/− mice had significantly reduced KC and IL-10 production, but many cytokines were unaffected versus WT mice (Figure 5B–D). IL-23p19 production trended towards decreased production in JNK1 −/− compared to WT mice (Figure 5E). Overall, these data show that JNK1 plays a minor role in lung inflammation induced by Influenza A, but is critical to determining morbidity and viral burden. One potentially key difference observed in JNK1 −/− mice by histopathology was the presence of plugging of airways (Figure 6A). This phenotype was not observed in any sections from WT mice. To determine if the airway plugging was perhaps due to mucus hyper-production, expression of Muc5ac, Muc5b, and Clca3 were examined. JNK1 −/− mice did not display different levels of mucin gene expression versus WT mice (Figure 6C). In addition, neither WT nor JNK1 −/− mice stained positive for mucus hyper-production by Periodic Acid Schiff staining (data not shown). Finally, the mechanism by which JNK1 −/− mice have lower Influenza A burden was investigated. The type I interferon response has been shown to be critical to improving viral host defense and clearance. WT and JNK1 −/− mice produced similar levels of IFNβ seven days after infection, suggesting no defect or enhancement of this pathway (Figure 7A). Since JNK1 has been shown to play a role in T cell survival, the impact of JNK1 deletion on T cell populations in the lung following viral infection was assessed. JNK1 −/− mice displayed similar ratios of CD4^+^, CD8^+^, γδT, and NKT cells as WT mice (Figure 7B). These data suggest that JNK1 −/− mice have appropriate T cell responses to Influenza A infection.

### JNK1 is required for IL-17A signaling in vitro and in vivo

**Figure 8:**
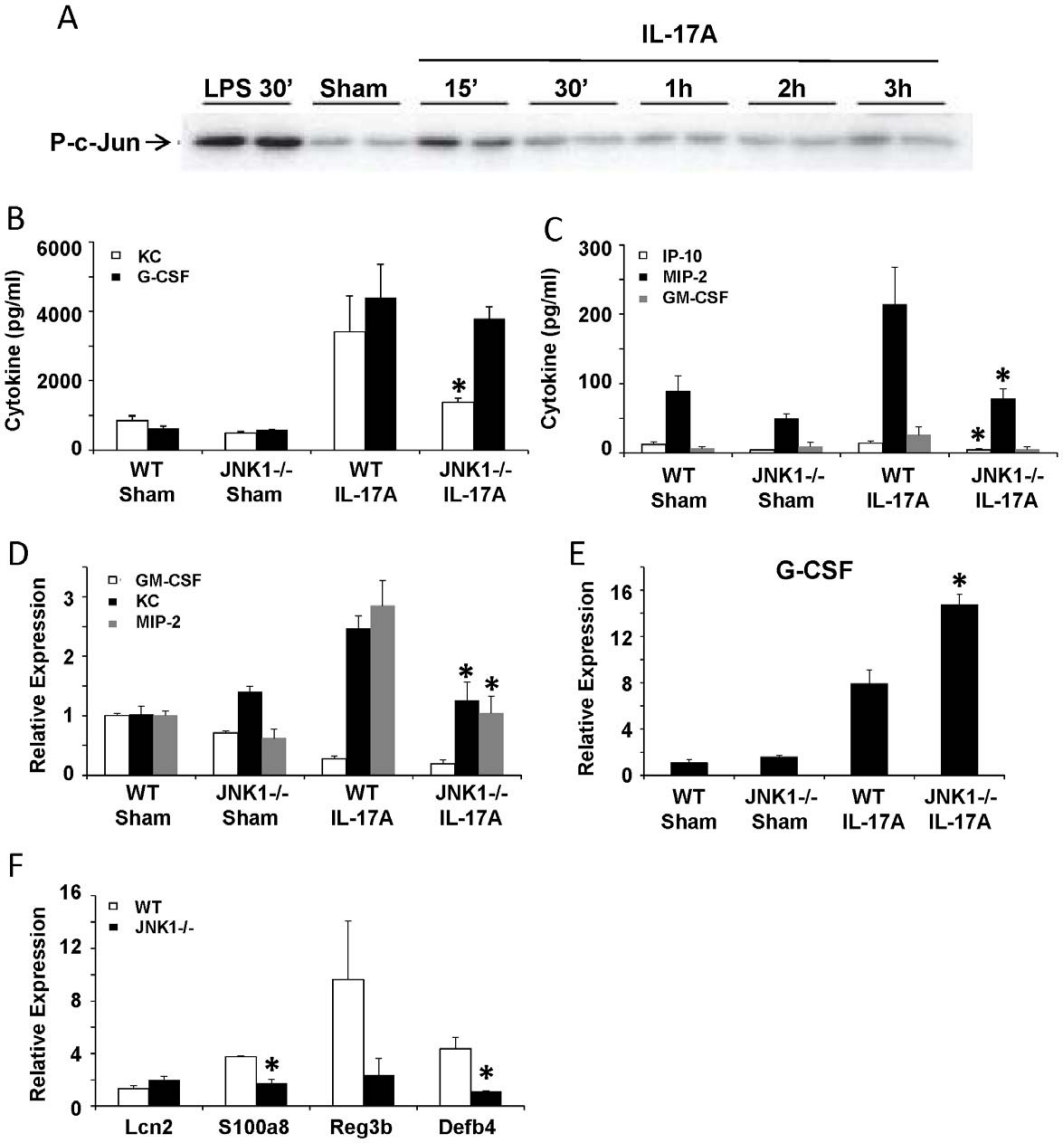
JNK1 is required for the epithelial cell response to IL-17A. MTEC from WT and JNK1 −/− mice were treated with IL-17A (10 ng/ml) for 24 hours. A – JNK1 activation by in vitro kinase assay. B, C – inflammatory cytokines produced (N = 6). D, E – inflammatory cytokine gene expression by RT-PCR (N = 3). F – antimicrobial peptide expression by RT-PCR (N = 3). * p<0.05.

The IL-17 pathway has recently been implicated in host defense against a number of both intra- and extra-cellular pathogens. IL-17A is known to be required for host defense and inflammation in response to gram-negative and gram-positive bacteria, as well as Influenza A infection. In models of bacterial pneumonia IL-17R signaling or IL-17A is required for pathogen clearance. In contrast in Influenza A infection, IL-17R signaling is dispensable for viral clearance, but is required for morbidity and lung injury [14]–[17]. Since JNK1 has a role in these infection paradigms and JNK1 −/− mice had a trend towards decreased IL-17A production, the role of JNK1 in IL-17A signaling was investigated. First, to confirm that IL-17A stimulates JNK1 activity, mouse tracheal epithelial cells (MTEC) were treated with IL-17A and JNK1 phosphorylation of c-Jun was determined (Figure 8A). IL-17A induced rapid activation of JNK1 as early as fifteen minutes after stimulation. IL-17A is known to stimulate inflammatory cytokine and antimicrobial peptide production by epithelial cells. WT and JNK1 −/− MTEC were stimulated with IL-17A for one day and cytokines were measured by multiplex cytokine assay and RT-PCR. IL-17A induced KC and MIP-2 protein and mRNA as well as decreased IP-10 protein were significantly decreased in JNK1 −/− MTEC compared to WT cells (Figure 8B–D). Surprisingly, JNK1 −/− MTEC had increased G-CSF mRNA, but no change in protein compared to WT cells, upon stimulation with IL-17A (Figure 8E). These data demonstrate that JNK1 is required for IL-17A pro-inflammatory signaling in vitro. In addition, JNK1 −/− MTEC expressed significantly decreased levels of the antimicrobial peptides S100a8 and Defb4 compared to IL-17A stimulated WT MTEC (Figure 8F). Taken together, the data suggest that IL-17A signals through JNK1 to induce inflammation and enhance host defense.

**Figure 9:**
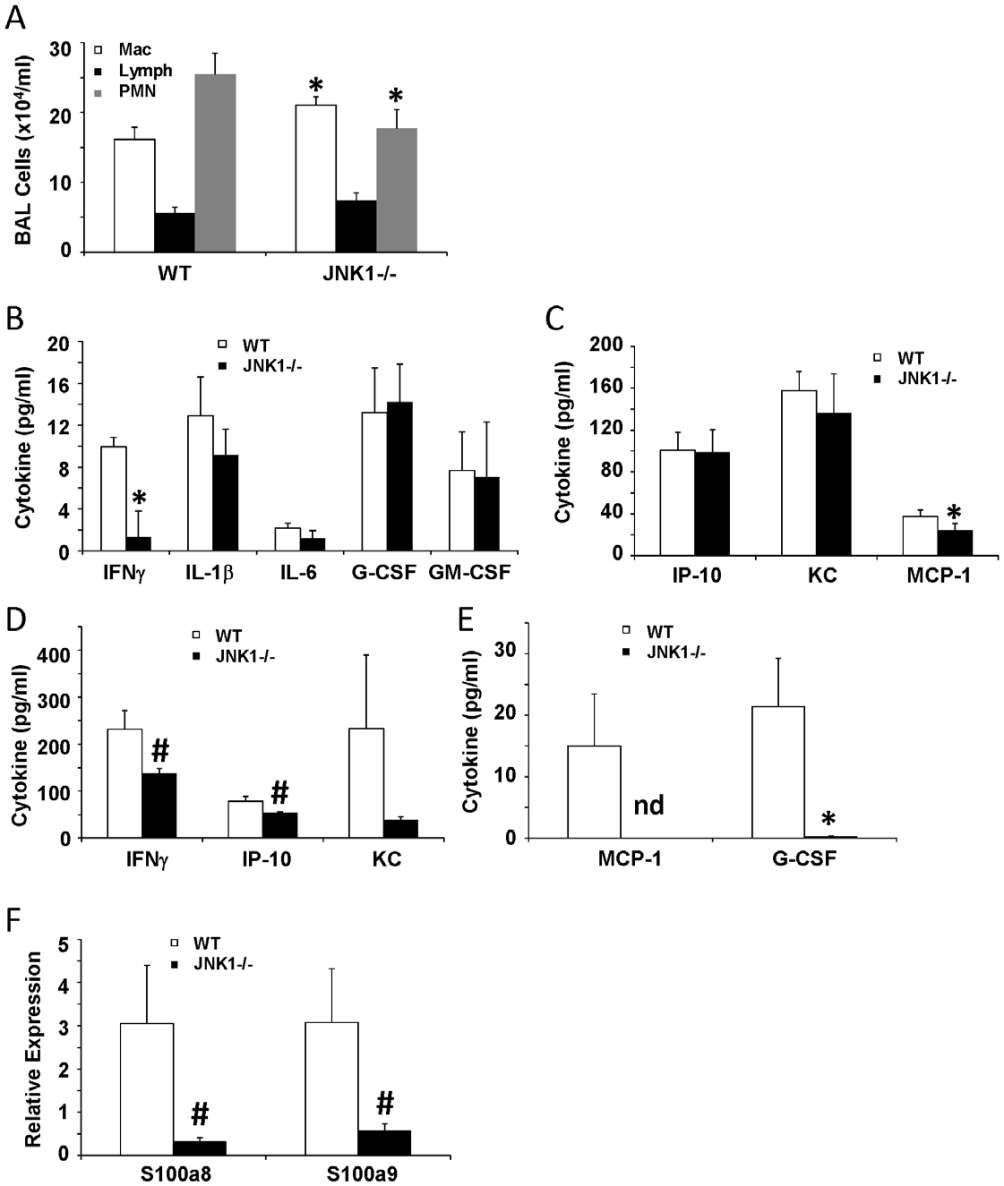
JNK1 modulates the inflammatory response to IL-17A in vivo. WT and JNK1 −/− mice were challenged with 5×10^8^ pfu of adenovirus expressing IL-17A for 3 days (A–C) (N = 8, 7) or 1 µg of recombinant IL-17A for 24 hours (D–F)(N = 6, 7). A – inflammatory cells in BAL fluid. B, C, D, E – inflammatory cytokines in lung homogenate. F – antimicrobial peptide expression in lung tissue. * p<0.05, # p<0.10, nd – not detected.

Since JNK1 was shown to play a role in IL-17A signaling in vitro in epithelial cells, the impact of JNK1 deletion on IL-17A signaling in vivo was investigated. WT and JNK1 −/− mice were challenged with adenovirus expressing IL-17A for three days. Adenoviral IL-17A induced similar levels of IL-17A protein in the lung; 4088.1±1069.5 pg/ml in WT mice and 4009.4±459.0 pg/ml in JNK1 −/− mice. The total numbers of inflammatory cells in the BAL were similar in WT and JNK1 −/− mice, however, JNK1 −/− mice had significantly increased macrophage and decreased neutrophil recruitment (Figure 9A). In addition to altered cellular infiltrate profiles, JNK1 −/− mice produced significantly decreased MCP-1 and IFNγ compared to WT mice (Figure 9B, C). The adenoviral expression approach utilized introduces the potential caveat of a differential viral response in the WT and JNK1 −/− mice. To further examine IL-17A signaling in vivo, WT and JNK1 −/− mice were instilled with recombinant mouse IL-17A for one day. IL-17A induced significantly decreased MCP-1 and G-CSF production, as well as a trend towards lower IP-10 and IFNγ, in JNK1 −/− mice versus WT mice (Figure 9D, E). Furthermore, JNK1 −/− mice stimulated with IL-17A demonstrated a trend towards decreased antimicrobial peptides S100a8 and S100a9 compared to WT mice (Figure 9F). These data show that IL-17A requires JNK1 for inflammatory signaling in vivo.

## Discussion

The results of this study indicate that JNK1 plays a context dependent role in host defense and inflammation. In general, JNK1 was associated with macrophage recruitment in response to each of the three pathogens tested. Furthermore, JNK1 was necessary for induction of MCP-1 and IFNγ, two important factors for macrophage function. JNK1 was also implicated in the production of antimicrobial peptides by epithelial cells and in the lung. These data suggest two potential mechanisms by which JNK1 may regulate host defense. In viral pneumonia, JNK1 had a somewhat paradoxical role, as JNK1 −/− mice had lower viral burden but worsened morbidity and lung histopathology. The mechanism for this did not appear to involve altered mucin gene induction based on the lack of impact on Clca3 mRNA, T cell recruitment, or type I interferon induction. Finally, JNK1 impacted IL-17A signaling in a similar manner to its effects on gram-negative bacterial pneumonia; decreased chemokine and antimicrobial peptide production. These data suggest that IL-17A requires JNK1 signaling which would suggest that JNK1 is required in a number of disease pathologies.

The impact of JNK1 in host defense against bacterial pathogens is largely unclear. Little is known about the impact of JNK1 deletion or inhibition in vivo. *Pseudomonas aeruginosa* induces JNK1 dependent apoptosis of cells via its exotoxin S, *E. coli* mediated induction of cytokines in HeLa cells was shown to be decreased by a JNK inhibitor, and LPS mediated increases of IL-23 was JNK1 dependent [18]–[20]. These data support the findings that JNK1 may be important in host defense against gram-negative bacteria. Our data indicate that JNK1 deletion has similar effects on *E. coli* and IL-17A induced cytokine production. Specifically, IFNγ and MCP-1 levels were reduced in JNK1 −/− mice challenged with both stimuli. These data suggest that JNK1 may play a role in macrophage function in host defense. *E. coli* has been previously shown to activate JNK1 in macrophages [21]. Furthermore, MCP-1 −/− mice fail to recruit neutrophils during E. coli pneumonia and have increase bacterial burden in the lung [22]. The link between IL-17A and *E. coli* pneumonia is supported by the findings that LPS activates IL-17A production in the lung and IL-17A −/− mice have increased *E. coli* burden in urinary tract infection [23]–[24]. In addition, RIP2 −/− mice have increased bacterial burden and decreased IL-17A production in the lung [25]. These data suggest that JNK1 may act downstream of IL-17A during *E. coli* pneumonia. The lack of an impact of JNK1 on host defense against gram-positive bacteria has not been previously reported. Peptidoglycan from *S. aureus* was shown to require JNK1 to drive IL-8 production in lung type II cells, suggesting a role for JNK1 [26]. Our data show a defect in macrophage recruitment but little impact on cytokine production.

Recent studies concerning JNK1 and Influenza A infection have focused on the ability of virus to inhibit JNK1 and thus alter host cell apoptosis [27]–[28]. JNK1 was shown to be inhibited via viral NS1 protein or host PI3K/AKT activity thus blocking apoptosis of infected cells. These data would suggest that in the absence of JNK1, viral burden may be increased due to a lack of apoptosis, however we observed decreased viral burden in JNK1 −/− mice. MLK3 −/− mice, a kinase upstream of JNK1, display increased Influenza A burden due to increased epithelial cell survival and viral replication [29]. The reason for the discrepancy with these data and our findings is unclear. Several studies have reported JNK1 activation following Influenza A infection [30]–[32]. In these studies Influenza A drove activation of JNK1, downstream AP-1 transcriptional activity, and cytokine production. Our data show that JNK1 deletion results in an altered inflammatory cellular phenotype in the lung and suppression of KC and IL-10 production. A recent microarray study with a JNK1 inhibitor showed decreased Influenza A induced IL-6 production, although in JNK1 −/− mice we did not observe this [33]#. Our data show that JNK1 −/− mice had increased numbers of lymphocytes in the BAL, but no change in the relative proportion of T cells versus WT mice. JNK1 has been shown to be required for CD8^+^ T cell proliferative responses to IL-2, via regulation of IL-2 receptor, CD25 [34]. A separate study showed that CD8^+^ T cell apoptosis requires JNK1 [35]#. These findings suggest opposing mechanisms by which JNK1 deletion would be expected to either increase or decrease CD8^+^ T cells in response to Influenza A. Our findings indicate a minimal effect on CD8^+^ T cell populations in the lung. At this time it remains to be determined why JNK1 −/− mice have lower viral burden, but worsened morbidity during Influenza A infection.

Interactions between the IL-17A and JNK1 signaling pathways have been recently described. In a number of diverse cell types, including airway smooth muscle cells and fibroblasts, IL-17A was shown to stimulate phosphorylation of JNK1 and promote cytokine production [36]–[44]. In these studies, pharmacologic inhibition showed that JNK1 is required for the IL-17A induced production of inflammatory mediators such as, IL-6, IL-8, eotaxin-1, and β-defensin 2. These data suggest that JNK1 is an important downstream signaling kinase in IL-17A induced inflammatory responses. Conversely, a few studies have failed to find a role for JNK1 downstream of IL-17A in epithelial cells [45]–[46]. A limitation of these studies is the use of pharmacological inhibitors which are somewhat non-specific and inhibit both JNK1 and JNK2. JNK1 likely impacts IL-17A signaling at the transcriptional level. AP-1 DNA-binding elements have been identified in the promoter regions of IL-17A-induced genes, including IL-6, KC, G-CSF, and MCP-1, indicating a potential target for JNK1 regulation [47]. The role of JNK1 within T cells is an active area of investigation. JNK1 has previously been shown to play a role in T_H_1/T_H_2 polarization and cytokine production [48], although its role in differentiation of T_H_17 cells is unknown. The impact of JNK1 deficiency with regards to IL-17A and airway epithelial cells was previously unclear. Our data show that JNK1 is required for induction of IFNγ, MCP-1, G-CSF and antimicrobial peptides. These data define a clear role for an IL-17A/JNK1 signaling axis in lung primary epithelial cells relevant to lung infection and in whole lung tissue.

The findings presented in this study indicate a diverse role for JNK1 in host defense in the lung. The potential role for JNK1 in regulation of macrophage responses in vivo is intriguing and requires further investigation. In addition, the role of JNK1 in regulating antimicrobial peptide production may have broad consequences in immunity against numerous extracellular pathogens. Finally, the impact of JNK1 on viral clearance and pathogenesis is intriguing and remains to be elucidated. Since JNK1 modulates some of the functional effects of IL-17A, it is likely that JNK1 is required for host defense in a number of TH17 mediated diseases. These data identify the JNK1 pathway as an important target in understanding lung immunity. Targeting JNK1 may provide a novel therapeutic approach for treating pneumonia.

## Materials and Methods

### Animals

Heterozygous JNK1 +/− mice on a N5 generation C57BL/6 background were purchased from Jackson Laboratories and were maintained as a breeding colony under pathogen free conditions. All experiments were conducted with age and sex matched JNK1 −/− and wild-type (WT) littermate controls. All animal studies were approved by the University of Pittsburgh Institutional Animal Care and Use Committee, protocol #0903113.

### Bacterial Infection Models

JNK1 −/− and WT mice were inoculated with *Escherichia coli* (DH5α, 10^7^ cfu) or *Staphylococcus aureus* (ATCC 49775, 10^8^ cfu) by oropharyngeal aspiration in 50 µl of sterile PBS. Bacteria were grown for 18 hours to stationary phase prior to inoculation. Twenty-four hours following infection, mice were lavaged with 1 ml sterile PBS for differential cell counts by cytospin. The right lung was then homogenized in 1 ml sterile PBS for bacterial colony counting, cytokine analysis by multiplex assay or by ELISA for IL-23p19, and real-time PCR for gene expression. The left lung was fixed in 10% neutral buffered formalin for histologic processing and H&E staining. Lung parenchymal and peribronchial inflammation were scored on double-blinded sections using a 0, least inflamed to 3, most inflamed. Each slide was scored twice and data reflect the cumulative inflammation score.

### Influenza A Infection Model

JNK1 −/− and WT mice were inoculated with Influenza A PR/8/34 H1N1 virus (150 pfu). Infected mice were then maintained for 7 days prior to harvest. Mouse lungs were lavaged and processed as detailed above. Viral burden was determined by viral plaque assay as previously described or by RT-PCR for viral matrix (M) protein expression [14]. T cell recruitment to the lung following Influenza A infection was determined by flow cytometry. Briefly, whole mouse lungs were digested with collagenase followed by mechanical separation. Lung cells were then stained with fluorescent conjugated antibodies to the surface markers CD4, CD8, γδTCR, and CD49b (DX5).

### Mouse Tracheal Epithelial Cell (MTEC) Culture

MTEC were prepared and propagated as previously described [3]. Cells were isolated from WT and JNK1 −/− mice and were maintained in submerged culture for studies with IL-17A. IL-17A was added to MTEC cultures at a concentration of 10 ng/ml for 24 hours or as indicated.

### JNK1 Kinase Assay

JNK1 activity in protein homogenates from MTEC was determined as previously described [4]. Briefly, JNK1 was immunoprecipitated from homogenates using anti-JNK1 antibody (Santa Cruz Biotechnology). JNK1 was then incubated with P32-ATP and a GST-c-Jun substrate for 30 minutes at 30°C. Phosphorylation of GST-c-Jun was then visualized by SDS-PAGE and autoradiography imaging.

### Exogenous IL-17A Models

Adenovirus expressing IL-17A was generated as previously described [16]#. WT and JNK1 −/− mice were instilled with 5×10^8^ pfu of adenovirus expressing IL-17A by oropharyngeal aspiration. Mice were then incubated for 3 days prior to harvest. Mouse lungs were lavaged and processed as described above. Additionally, WT and JNK1 −/− mice were instilled with 1 µg of recombinant mouse IL-17A for 24 hours prior to similar lung processing.

### Statistics

Data were analyzed by unpaired two-tailed *t*-test or by one-way ANOVA where appropriate. For multiple comparisons, following ANOVA, data were compared by Tukey test. Analyses with a resultant p<0.05 were determined significant, additionally p<0.10 is also reported as a trend. Data are presented as mean ± standard error of the mean. All studies were repeated at a minimum of two times with the resultant combined data presented, except for MTEC gene expression data where representative data is shown. All analyses were conducted with the Microsoft Excel software package.
